# Worse Than the Wicked Flea

**Published:** 1869-10

**Authors:** 


### WORSE THAN THE WICKED FLEA."

"They tell us of the Egyptian asp,
The sting of which is death;"

And the Divine revelator saw a vision of locusts that "had tails like unto scorpions, and there were stings in their tails; and their power was to hurt men five months." But it has been reserved for these later days, and for this newest profession to note an event in the way of stinging, so terrible and so prolonged in its effects, that these oriental pests, whether of nature or revelation, are thrown into the shade, deeper than was the sun by the late eclipse. Nor do we speak of the tarantula; for tradition tells us that its poison is eliminated by the dancing of the victim. But in the melancholy cases under consideration, though the poor patients have frisked around considerably, they are not in the least improved, but the symptoms are quite as malignant as when the sting was fresh. Worse than the asp, then; for death soon comes to the rescue. Worse than the locusts; for "their power was to hurt men five months," while the venemous monster under contemplation seems to have power to hurt almost as many years. And, stranger than all, it has been regarded as perfectly harmless, till the occurrence of the sad cases referred to.

Those who attended the Chicago meeting of the American Dental Association, in 1865, and who read the journals afterward, will recollect that the two members who carried on the meeting were stung by a drone—stung, too, while they were kindly feeding him on "royal jelly," for the purpose of changing his sex, that he might be crowned queen of the hive, and become the happy mother of a countless progeny. As in many other cases of blood poisoning, the sting seemed to pervert both the mental and moral characters of the victims. It was difficult for them to refrain from talking nonsense, and apparently still harder to tell the truth, especially when speaking of a friend who tried harder, perhaps, than any other to remove the sting. And, now, after a lapse of four years, these symptoms are manifested as strong as ever, as we have learned from a recent number of the *Dental Cosmos*. In their ravings they speak of one feeble individual "forcing" an entire association, making out a fearful case of rape, unparalleled, and likely to remain so till some diabolical dwarf outrages an army of Amazons.

In many cases of chronic blood poisoning the victims become cowardly; but these poor fellows are brave. They are not afraid to attack a man who is a thousand miles away, especially if they know he will not find it out for a month or two, and more especially if they think he is likely to stay away. And they find it as hard as ever to tell the truth about him. Indeed they have made a total failure, if the report is correct, and it probably is; for the reporter is a prophetic character, described in the second clause of the eighth verse of the seventh chapter of Daniel, as follows: "Behold there came up among them another little horn." Being more familiar with slang than with science, he has been careful to preserve all their twaddle, regardless of its personal and unparliamentary character. We could not expect even a prophetic reporter to omit anything tending to injure the reputation of one he had been hired to villify when supposed to be dying. Hence we have no complaint. As "Nip" is dead, "Snarler" must be allowed to growl and snap, to his heart's content.

